# Novel Factors Regulating Proliferation, Migration, and Differentiation of Fibroblasts, Keratinocytes, and Vascular Smooth Muscle Cells during Wound Healing

**DOI:** 10.3390/biomedicines12091939

**Published:** 2024-08-23

**Authors:** Jacob Smith, Vikrant Rai

**Affiliations:** Department of Translational Research, Western University of Health Sciences, Pomona, CA 91766, USA; jacobsmith@westernu.edu

**Keywords:** diabetic foot ulcer, wound healing, angiogenesis, ECM remodeling, cell migration, cell proliferation

## Abstract

Chronic diabetic foot ulcers (DFUs) are a significant complication of diabetes mellitus, often leading to amputation, increased morbidity, and a substantial financial burden. Even with the advancements in the treatment of DFU, the risk of amputation still exists, and this occurs due to the presence of gangrene and osteomyelitis. Nonhealing in a chronic DFU is due to decreased angiogenesis, granulation tissue formation, and extracellular matrix remodeling in the presence of persistent inflammation. During wound healing, the proliferation and migration of fibroblasts, smooth muscle cells, and keratinocytes play a critical role in extracellular matrix (ECM) remodeling, angiogenesis, and epithelialization. The molecular factors regulating the migration, proliferation, and differentiation of these cells are scarcely discussed in the literature. The literature review identifies the key factors influencing the proliferation, migration, and differentiation of fibroblasts, keratinocytes, and vascular smooth muscle cells (VSMCs), which are critical in wound healing. This is followed by a discussion on the various novel factors regulating the migration, proliferation, and differentiation of these cells but not in the context of wound healing; however, they may play a role. Using a network analysis, we examined the interactions between various factors, and the findings suggest that the novel factors identified may play a significant role in promoting angiogenesis, granulation tissue formation, and extracellular matrix remodeling during wound healing or DFU healing. However, these interactions warrant further investigation to establish their role alone or synergistically.

## 1. Introduction

Diabetes mellitus is a rapidly growing chronic disease affecting 422 million people worldwide [1]. Diabetes is a metabolic disease characterized by hyperglycemia. Type 1 and type 2 are the two major types of diabetes mellitus. Type I diabetes mellitus (DM I) is classified by a lack of proper insulin production in the pancreas due to the destruction of insulin-producing pancreatic beta cells and it is normally first presented in adolescence, while type 2 diabetes (DM II) is characterized by decreased insulin secretion from the pancreas or the inability to use the secreted insulin, termed insulin resistance, due to prolonged hyperglycemia. Roughly 15–25% of those diagnosed with diabetes will develop diabetic foot ulcers at some point in their lifetime, resulting in between 9 and 25 million patients worldwide [2,3]. According to the Centers for Disease Control and Prevention, 60–80% of all lower limb amputations in the United States were directly as a result of diabetes [4].

Diabetic foot ulcers (DFUs) are an example of a chronic wound or a wound that does not progress through the normal stages of wound healing and becomes halted in the inflammatory phase without progressing to the resolution phase. For a wound to properly heal it must progress through four distinct wound-healing stages, which are hemostasis, inflammation, proliferation, and remodeling [5]. Immediately after the wound occurs, hemostasis begins the cessation of bleeding from a blood vessel. The two major functions of hemostasis are the clotting and release of growth factors. During hemostasis, platelet activation results in an increased secretion of cytokines, including transforming growth factor-β (TGF-β) and platelet-derived growth factor (PDGF). This occurs to promote the chemotaxis of neutrophils and macrophages initiating the inflammatory phase [6,7], the prolongation of which results in a nonhealing state of DFU [8,9]. Due to the chronicity of the inflammation contributing to the nonhealing of DFU, the advancements in wound healing methods, including wound dressings to maintain moisture and control the exudate, off-loading the ulcer of necrotic tissue, medication, and infection prevention, chronic DFUs remain an expensive and deadly issue [10]. Thus, to promote wound healing, the first step should be to attenuate the inflammation and then promote the infiltration of fibroblast, keratinocytes, and endothelial cells for ECM synthesis and remodeling, followed by angiogenesis playing a critical role in wound healing [11,12].

The role of targeting inflammation and fibroblast phenotype change [8,10,13,14], using stem cells to promote angiogenesis [14], and various factors contributing to or regulating inflammation, angiogenesis, ECM remodeling, and wound healing have been discussed [11,12,15]; however, the factors regulating the recruitment of fibroblasts, endothelial cells, vascular smooth muscle cells, and keratinocytes to the site of injury are not yet well discussed. Growth factors play a critical role in regulating the proliferation and migration of various cell types, including keratinocytes, fibroblasts, endothelial cells, and vascular smooth muscle cells [16,17,18,19,20], inflammation, and angiogenesis. The results of the studies using growth factors to promote wound healing are encouraging but still there is a need to find better therapeutics. This review aims to first comprehensively describe wound healing and the growth factors discussed in the literature, followed by describing the novel factors, which have not been discussed in the literature in regard to wound healing, regulating the proliferation and migration of epithelial cells (keratinocytes), fibroblast, endothelial cells, and vascular smooth muscle cells (VSMCs). These novel factors may be used in combination with the existing treatment to improve wound healing strategies.

## 2. Normal Wound Healing

**Figure 1:**
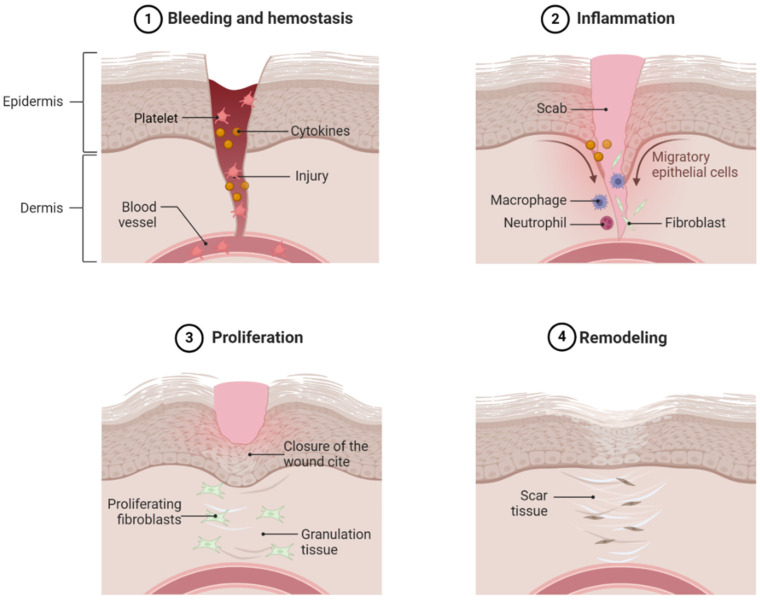
Physiological wound healing. Normal dermal wound healing consists of four phases, namely hemostasis (**1**), inflammation (**2**), proliferation (**3**), and remodeling (**4**). Alteration in any event, persistent inflammation, decreased fibroblast and keratinocyte infiltration in the wound area, and decreased ECM remodeling and angiogenesis contribute to nonhealing diabetic foot ulcers. Hyperglycemia is a major underlying pathophysiology of these events.

After hemostasis, the next phase of wound healing is the inflammatory phase (Figure 1). The inflammatory response is triggered by activated platelets, cytokines, and the products of hemostasis [21]. Many cells play a crucial role in the inflammatory process. In the early response, neutrophils arrive on the scene first. Their primary function is to kill various microbes to prevent infection. After the bacteria and debris have been cleaned by the neutrophils, they are removed from the wound site by apoptosis. Another key feature of the inflammation phase is the release of pro-inflammatory cytokines and chemokines at the wound site, which are crucial in the process of wound healing. Cytokines, such as interleukin (IL)-1, IL-6, and tumor necrosis factor (TNF)-α, work together to activate immune cells, regulate epithelial and fibroblast cells, and prevent infection [22].

The major focal point of the proliferative phase after the inflammation phase is the closure of the wound surface and the restoration of skin function (Figure 1). During proliferation, wound closure is achieved through keratinocyte activation, resulting in the formation of a protective epithelial barrier [23]. The preexisting keratinocytes at the wound edge begin to generate more cells to cover the wound. These keratinocytes rebuild the basal membrane through protein secretion. During the process of proliferation, the wound is replaced by granulation tissues, a complex of collagen bundles, composed of macrophages, granulocytes, fibroblasts, and blood vessels [24]. One cell that plays a key role in the formation of granulation tissue is the fibroblast. Fibroblasts, unique spindle-shaped cells, are mesenchymal-derived cells that produce collagen and the extracellular matrix (ECM). They play a diverse role in intracellular wound healing, primarily functioning to promote angiogenesis and construct and reshape the ECM [25].

The final step in wound healing is the remodeling phase (Figure 1). After the development of granulation tissues in the proliferation phase, the remodeling phase begins, where the wound matrix breakdown is relegated by fibroblasts. The goal of this is to achieve tensile strength and recover normal tissue structures. This stage is also critical for the recovery of the “normal” tissue appearance. This results from a decreased secretion of pro-inflammatory cytokines and an increased secretion of anti-inflammatory cytokines, such as IL-10 or transforming growth factor (TGF)-β1, helping in tissue remodeling. Collagen synthesis during ECM formation and remodeling is regulated by a plethora of growth factors, such as TGF-β1 and fibroblast growth factor (FGF), which have a strong effect on genic expression [24,26,27]. Another key feature of the remodeling stage is the selective reduction of angiogenesis, which is crucial in proper wound healing [28]. During wound healing, interactions from fibroblasts, keratinocytes, and endothelial cells utilize paracrine effects to regulate wound healing.

## 3. Role of Keratinocytes, Epithelial Cells, and Fibroblasts in Wound Healing

Three major cell types that play critical roles in wound healing are fibroblasts, keratinocytes, and VSMCs. Many studies have indicated that fibroblasts are essential in the treatment of diabetes and its complications. Studies have indicated that fibroblasts play many roles in health and diseases, beyond being just the immune-neutral cells they were originally classified as. Their key feature in wound healing is to construct and reshape the ECM. They detect injury and pathological stimulation to activate and regulate immune responses [25]. Further, the fibroblast heterogeneity and the effect of the wound microenvironment on fibroblasts also play a critical role in wound healing [12]. One study identified that during wound re-epithelialization, fibroblasts may migrate to the epicenter of the wound. Interactions between fibroblasts, keratinocytes, and endothelial cells utilize paracrine effects to regulate wound healing. One major characteristic of fibroblasts is their ability to change phenotype, altering their functionality. In the presence of a diabetic ulcer, the wound microenvironment is altered, due to the secretion of various cytokines (TGF-β, IL-6, IL-8), resulting in phenotypic changes in fibroblasts, affecting wound healing [12]. Fibroblast proliferation and migration are negatively regulated by ECM proteins [29].

Key fibroblast phenotypes are quiescent fibroblasts, myofibroblasts, and secretory fibroblasts. Quiescent fibroblasts, the largest population of dermal fibroblasts (anti-CD34+, anti-HSP47+, anti-SFA+, S100A4+), are common during the normal physiological state and the fibrosis phase. During the inflammation and resolution phases of wound healing, fibroblasts activate and are transdifferentiated to myofibroblasts (α-SMA+, fibronectin+, cadherin+, n-caldesmon−, smoothelin−, and desmin−) with the characteristics of both fibroblasts and smooth muscle cells [12]. These myofibroblasts contribute to wound healing by promoting ECM remodeling and tissue formation. During the early stages of wound healing, myofibroblasts actively migrate toward the wound bed to mediate ECM deposition and remodeling. The transitioning of fibroblasts to myofibroblasts, and then to quiescent fibroblasts, is controlled by both cellular and molecular mediators [30]. Studies show that the regulation of cytokine levels and fibroblast phenotype in the wound microenvironment may lead to a significant increase in the wound healing of diabetic foot ulcers [12]. Myofibroblasts also contribute to wound contraction through the activation of contractile signaling pathways involving non-muscle myosin II, ACTB, and ACTG1 [31].

Secretory fibroblast (CD 40+) density significantly increases in diabetic foot ulcers, which contributes to chronic inflammation [13]. The literature indicates that decreased populations of myofibroblasts and increased populations of secretory fibroblasts may lead to the nonhealing of DFUs. The phenotype shift to CD-40+ results in an increased secretion of IL-6 and IL-8. The increased expression of IL-8 has been associated with nonhealing DFUs. This correlation indicates that phenotype shifts to secretory fibroblasts play a role in nonhealing DFUs [30]. Thus, decreasing the secretory fibroblast population in a nonhealing DFU may result in amplified wound healing by attenuating the levels of pro-inflammatory cytokines and decreasing the phenotypic switch of the fibroblasts. Additionally, the role of angiogenic (FSP1+/CD31−/CD45−) and angiostatic (TSP1+) fibroblasts with their role in cardiac remodeling after infarction has been discussed in the literature but their role in the context of DFU healing is not well established [12].

Keratinocytes are the most predominant epithelial cell type [32]. They play crucial roles in normal skin function, such as the origination of the basal layer, keratin production, lipid secretion, and calcium absorption. Keratinocytes play a key role in wound healing, restoring the epithelial barrier. Wound microenvironment cues, such as cytokines, growth factors, and chemokines, give rise to the different cellular states of keratinocytes. Keratinocytes secrete cytokines and chemokines to recruit, activate, and regulate immune cells. dsRNA, coupled with the cytokines present at the wound site, activates dsRNA-sensing receptors in keratinocytes, resulting in a positive feedback loop, which leads to an immune response. Keratinocytes also express Atp-binding cassette sub family A member 12 (*ABCA12*) and transglutaminase 1 (*TGM1*), which are genes that are essential for skin repair and maintenance [33].

Keratinocyte fibroblast interactions have also been shown to play an important role in wound healing. The evidence suggests that keratinocytes promote the synthesis of growth factors by fibroblasts, which, in turn, stimulate keratinocyte proliferation in a double paracrine manner [34]. This interplay results in a positive feedback mechanism that is critical for effective wound repair. One factor that is upregulated in keratinocytes as a result of skin injury is transforming growth factor alpha (TGF-α). Predominantly expressed in keratinocytes, TGF-α exerts potent autocrine effects, significantly enhancing keratinocyte activity. High TGF-α expression has also been shown to be analogous with keratinocyte hyperproliferation [34]. Keratinocyte and fibroblast interactions have been shown to contribute to re-epithelialization. Epidermal keratinocytes have been hypothesized to alter fibroblast activity via cytokine connective tissue growth factor (CTGF). CTGF is a regulatory protein, which promotes the proliferation of mesenchymal cells, such as fibroblasts [35]. Transforming growth factor β1 (TGF-β1) stimulates the expression of CTGF, though CTGF may also act independently. Both TGF-β1 and CTGF are upregulated during wound healing and may be overexpressed in different fibrotic conditions [35].

The migration of keratinocytes is another essential component of wound healing, facilitating the restoration of the disrupted physical barrier between the wound site and the external environment. The reformation of the epidermis is achieved through the stratification of keratinocytes, which are interconnected via adherens junctions. These junctions are crucial, as they anchor the actin cytoskeletons and plasma membranes of adjacent cells [36]. The presence of diabetes results in a long-term, high-glucose environment, negatively affecting wound healing. Patients with diabetes have reduced levels of proliferation and migration of keratinocytes. This decreased proliferation and migration of keratinocytes results in insufficient re-epithelization of the wound, negatively altering the process of wound healing [25].

The most abundant cell type in vessels is VSMCs, which have a high plasticity. When a vessel injury occurs, VSMCs differentiate cell types from synthetic VSMCs to have increased migration and proliferation capabilities [37]. VSMCs can also change the phenotype, and the data indicate that the phenotype shift of VSMCs might be impacted by proinflammatory cytokines released by M1 macrophages. VSCM plasticity and phenotype shift have been shown to play a role in the regulation of atherosclerosis, plaque progression, vessel wall inflammation, and adverse remodeling [38]. Research indicates that the migration of VSMCs to the wound site may be a result of exposure to growth factors. Injury plays a key role in the growth dynamics of SMCs, increasing the rates of migration and proliferation to aid in wound healing [39].

Vascular endothelial cells and vascular lymphatics also play curtail roles in wound healing. In normal tissue, vascular endothelial cells regulate blood flow, control the permeability of the vessel wall, and maintain the fluidity of the blood. In response to the inflammatory phase of an injury, the recruitment of neutrophils activates endothelial cells. Vascular endothelial cells undergo phenotype changes, aiding in many phases of the inflammatory process [40]. There are two major types of endothelial activation: type 1 activation, which is independent of new gene expression, and type 2 activation, which is slower and depends on gene expression. Type 1 activation is controlled by ligands binding to G-protein coupled receptors, while type 2 activation is a sustained activation, mediated by tumor necrosis factor [40]. The restoration of skin function in wound healing is aided by the process of lymphangiogenesis; the formation of new lymphatic vessels [41]. Current research aims to identify the relationship between lymphangiogenesis and diabetic wound healing. Lymphangiogenesis has been shown to play a role in the inflammatory response. The loss of lymphatic vessels may induce inflammatory environments, which results in delays in wound healing [42].

## 4. Factors Regulating the Proliferation and Migration of Keratinocytes, Fibroblasts, and Smooth Muscle Cells

Growth factors have been shown to play a critical role in regulating various molecular aspects during wound healing, including the proliferation and migration of keratinocytes, fibroblasts, and VSMCs in normal physiological healing and diabetic foot ulcer healing (Table 1). The studies [16,17,18,19,20] performed using an in vitro model and an in vivo model with an animal model suggest the important roles of growth factors in promoting wound healing in DFU; however, only a few clinical trials have been conducted using these growth factors to show enhanced DFU healing in human patients (listed in Table 2).

Insulin-like growth factor 1 (IGF-1) is a peptide hormone that stimulates the proliferation of keratinocytes. It initiates cell spreading and membrane protrusion. IGF-1 stimulates phosphatidylinositol-3-kinase, which is necessary to induce cell-shape changes in keratinocytes [43]. IGF-1 levels are decreased at the wound site, indicating that its absence may play a role in delayed wound healing [44]. IGF1 promotes keratinocyte migration, promoting epithelialization and the contraction of the wound bed [45]. The beneficial role of IGF-1 is supported by increased wound healing with IGF-1 via the activation of IGF1R, promoting wound re-epithelialization, the epithelial tissue area, granulation tissue formation, and angiogenesis [46,47].

Epidermal growth factor (EGF), a polypeptide involved in epithelial maturation, initiates the mitogen-activated protein kinase (MAPK) pathway, and together with IGF-1, it can influence keratinocyte migration, which determines the wound epithelialization speed [43]. EGF significantly improves the healing rate of DFUs; however, it is important to regulate blood sugar during treatment with EGF [48,49]. EGF increases the number of fibroblasts and angiogenesis to promote wound healing [50]. EGF treatment decreases the expression of inflammatory mediators and increases the expression of the mediators involved in cell proliferation, angiogenesis, and ECM secretion [51]. The potential therapeutic role of EGF in enhancing wound healing and the decreased incidence of amputation has been documented [52]. These results suggest that EGF promoted DFU healing but most of the studies have described the topical use of EGF and more studies are warranted on the interstitial or intradermal use of EGF [53].

Fibroblast growth factor (FGF) is a polypeptide growth factor, with 23 different variants. FGF stimulates the proliferation of fibroblasts and angiogenesis, contributing to the formation of granulating tissues. FGF subtypes, such as aFGF, bFGF, and FGF 15/19, have been shown to affect the healing of wounds in diabetic conditions [54]. Further, a significant reduction in cure time and increased healing response after topical FGF application [53] support the notion that FGF has a beneficial effect on DFU healing by promoting angiogenesis, granulation tissue formation, re-epithelialization, and the detoxification of reactive oxygen species involving fibroblasts and keratinocytes and ERK2, Nrf2, Nrf3, and peroxiredoxin-6 signaling [16]. These studies suggest that FGF enhances wound healing by keratinocytes and fibroblast proliferation, contributing to angiogenesis, ECM formation and remodeling, and re-epithelialization [55,56,57]; however, an RCT concluded with low-quality evidence that EGF, platelet-rich plasma, and PDGF therapy promote wound healing, but no comment was related to FGF [58]. Thus, more clinical trials/studies are warranted on the efficacy of FGF.

Transforming growth factor (TGF)-β, a multifunctional cytokine, is expressed by most of the tissue and cell type. During acute wound healing, TGF-β is secreted by keratinocytes, suppressing the secretion of TGF-β from fibroblasts, which is required to revert the keratinocytes to their basal phenotype that is dose-dependent. TGF-β promotes keratinocyte proliferation, migration, differentiation, and the phenotypic switch, involving integrins and Smad signaling [59,60]. Further, the interaction between keratinocytes and fibroblasts is a must for skin homeostasis and wound healing [61]. Among the various isoforms of TGF-β, TGF-β3 induces regenerative characteristics in dermal fibroblasts [62], while TGF-β1 promotes proliferation and migration during wound healing [63]. TGF-β1 maintains skin homeostasis by inhibiting keratinocyte proliferation, regulating keratinocyte differentiation, and regulating the functions of keratinocytes and fibroblasts among other cells during wound healing. TGF-β1 promotes keratinocyte proliferation and migration during wound healing to promote re-epithelialization and fibroblast recruitment to the wound site [64,65]. These studies suggest that TGF-β plays a critical role in keratinocyte and fibroblast proliferation and migration during wound healing [59,64,66].

Platelet-derived growth factor-BB (PDGF-BB) is another growth factor that is one of the first released in the wound environment by activated platelets/degranulation, monocytes/macrophages, fibroblasts, and endothelial cells. PDGF-BB promotes granulation tissue formation and re-epithelialization by promoting fibroblast and keratinocyte proliferation and migration, contributing to enhanced wound healing when delivered in combination with VEGF, EGF, and IGF [67]. Additionally, PDGF also regulates mitogenic and chemotactic activity in fibroblasts and smooth muscle cells and the proliferation and differentiation of endothelial cells. This suggests that PDGF contributes to promoting wound healing by promoting ECM synthesis and angiogenesis [68]. This is supported by the findings of increased collagen deposition, angiogenesis, and enhanced wound healing using a PDGF-BB-derived hydrogel via stimulated fibroblast proliferation and migration [69,70]. The role of PDGF in promoting wound healing is further supported by the fact that in the initial phases of wound healing, PDGFRα signals the proliferation of fibroblast progenitors for fibroblast proliferation, while in the later phase, the downregulation of PDGFRα contributes to fibroblast differentiation into myofibroblasts that are necessary for ECM [71].

The studies discussed above suggest an important role of various growth factors in regulating fibroblast and keratinocyte proliferation and migration contributing to enhanced wound healing. This notion is also supported by various other studies (Table 2), as well as a meta-analysis of eight randomized clinical trials [48] concluding that EGF promotes wound healing in DFU. Another meta-analysis and systemic review including 281 studies concluded that the growth factors EGF, FGF, and GM-CSF increase the healing rate of acute skin wounds and decreased scar scores [72]. However, among all these growth factors, only PDGF has been approved by the FDA (Regranex; becaplermin gel, a topical gel for chronic wound) [73] for the treatment of chronic wounds and diabetic neuropathic ulcers. The various aspects of the failure of growth factors to be approved for wound healing have been discussed elsewhere [74].

It is important to note that only a few studies have performed a clinical trial using these growth factors promoting wound healing in DFU; however, there is indirect evidence to show the role of various growth factors in promoting wound healing in DFU. For instance, significantly increased IGF-1 levels in healed DFU after hyperbaric oxygen therapy suggest the role of IGF1 in promoting wound healing in DFU [75]. Other studies involving human patients (marked with *) have been listed in Table 2. These growth factors showed improved wound healing in DFU, but among EGF and PDGF, only PDGF received an FDA approval for DFU treatment, as mentioned above.

**Table 2:** Recent evidence to support the use of growth factors in promoting wound healing.

Growth Factors	Type of Study/Model	Outcome of the Study	Mechanism/Observation
IGF1 [76]	HUVEC cells and C57BL/6 mice	Promotes angiogenesis, attenuates inflammation, and enhances wound healing	Via Ras/PI3K/IKK/NF-κB signaling pathways
IGF-1 with HBOT [75]	48 patients with DFU treated with HBOT	HBOT increases IGF-1 expression and promotes wound healing	Increased IGF-1 with HBOT
IGF1 + Silk Fibroin [47]	Cg-*Dock^7m^* ^+/+^ *Lepr^db^*/JNarI female mice (db/db)	Promotes wound healing in diabetic mice	Increased the epithelial tissue area and microvessel formation
* hEGF [77]	Randomized clinical trial of 61 human patients with DFU	0.04% [*wt*/*wt*] hEGF is more effective in promoting wound healing and reducing healing time compared with 0.02% [*wt*/*wt*] hEGF	The effects of Actovegin plus 0.02% (*wt*/*wt*) hEGF and Actovegin plus 0.04% (*wt*/*wt*) hEGF on DFU healing was evaluated
* EGF-CMC [78]	Randomized clinical trial of 25 human patients with DMII and DFU	EGF-CMC is better in the colonization of strains producing less biofilm compared with CMC alone	Colonization of wounds by strains with lower biofilm formation abilities
* EGF-NPWT [79]	Retrospective study of 286 DFU patients	Decreases the rate of amputations in DFU	EGF alone or in combination with NPWT reduces amputation numbers
Human rFGF [80]	Primary human dermal fibroblasts	FGF improves fibroblast proliferation	Chemoattractant for fibroblasts
FGF2 [81]	C57BL6/J mice	Promotes epithelial–mesenchymal transition by downregulated E-cadherin and upregulated vimentin expression	Increased keratinocyte proliferation and migration
FGF2 [82]	Primary human dermal fibroblasts C57Bl/6 NRj female mice	FGF2 promotes proliferation and migration, and improves wound healing	Dermal fibroblast (DF)-derived extracellular vesicles (EVs) stabilize FGF2
* PDGF [83]	922 patients with a full-thickness diabetic neurotrophic foot ulcer	100 μg/g PDGF significantly increases complete healing. PDGF also decreases the time to complete healing by 30%.	Enhanced wound healing with PDGF gels
* recombinant human PDGF [84]	28 patients with DM II and chronic DFU	PDGF enhances DFU wound healing	rhPDGF 0.01% gel at a dose of 2.2 µg/cm^2^/day for 12 weeks
* PDGF-B (GAM501) Phase 1/2 clinical trial	15 patients with nonhealing neuropathic diabetic foot ulcers	GAM501 was safe and well tolerated. GAM501 enhances wound healing in DFU.	Four administrations of GAM501 at 1-week intervals (locally covering the wound area) were administered to patients
Peptide-derived PDGF-BB [85]	L929 fibroblast cells Wistar Albino Rats	Promotes wound healing	Increased fibroblast migration. Increased granular tissue formation, re-epithelialization, angiogenesis, and collagen formation.
Peptide-derived from PDGF-BB [86]	Multiple cell lines BALB/c female mice	Promotes wound healing	Increased cell proliferation (fibroblasts and keratinocytes)
EVs with IFG and TGF-β [87]	Multiple cell lines Human patients	Promotes wound healing	Increased fibroblasts, endothelial cells, and mesenchymal stem cell proliferation. Activation of Akt and ERK.
VEGF-A, PDGF-BB, HB-EGF [67]	NOD mouse model	Promotes wound healing in type 1 DM	Changes the cellular milieu of the wound environment
VEGF-A and FGF1 [88]	B6.BKS(D)-Leprdb/J (db/db mice)	Improves wound healing	Increased neovascularization
mesoglycan/VEGF [89]	C57Bl6 mice	Increased deposition of granulation tissue	Induces angiogenesis. Increased fibroblasts recruitment.

Insulin-like growth factor (IGF)1, human umbilical vein endothelial cells (HUVECs), phosphoinositide 3-kinases (PI3Ks), nuclear factor kappa B (NF-κB), epidermal growth factor-loaded carboxymethylcellulose (EGF-CMC), negative pressure wound therapy (NPWT), diabetic foot ulcer (DFU), fibroblast growth factor (FGF), platelet-derived growth factor (PDGF), transforming growth factor beta (TGF β), vascular endothelial growth factor (VEGF), hyperbaric oxygen therapy (HBOT), and heparin-binding EGF-like growth factor (HB-EGF). * indicates the growth factors investigated in human patients to promote wound healing.

Vascular smooth muscle cells (VSMCs) contribute to angiogenesis during wound healing and proliferation and the migration of VSMCs towards the wound site is important and is regulated by various factors. The phenotypic switch of VSMCs, which affects both proliferation and migration, plays a critical role during wound healing, angiogenesis, vessel remodeling, and tissue regeneration [11,12,28,90,91]. In type 2 diabetes, mitochondrial Ca^2+^ regulates the proliferation of VSMCs [92]. VSMC proliferation is regulated by SRSF1 (serine/arginine-rich splicing factor 1) after vascular injury [93] and the NF-κB p65/microRNA-17/RB pathway activation during inflammation [94]. MMP-2 and MMP-9 regulate VSMC migration and proliferation by degrading the matrix and non-matrix substrates [95]. The proliferation and migration of VSMCs are also regulated by various growth factors and cytokines, including angiotensin II, basic FGF, EGF, IGF-1, IL-6, IL-1β, PDGF (mainly PDGF-BB isoform), TGF-β1, VEGF secreted by ECs, and TNF-α [96,97,98,99]. TGF-β inhibits PDGF-induced VSMC proliferation via cyclin D1 downregulation, involving Akt-dependent Smad signaling after vascular injury [100], while basic FGF stimulates quiescent VSMCs and enhances proliferation and migration [101]. Antioxidant protein peroxiredoxin 1 promotes VSMC proliferation and migration involving TLR-4 [102]. These studies suggest that the proliferation and migration of VSMCs are regulated not only by growth factors but also by mediators involved in inflammation and oxidative stress, the factors playing a critical role in DFU pathogenesis.

The proliferation and migration of keratinocytes, fibroblasts, and VSMCs play a critical role in DFU healing and these are regulated by various factors. However, despite knowing these facts, there is still a need for better treatment strategies to promote healing, because of the risk of amputation due to chronic nonhealing ulcers. This suggests the need to delineate other factors, in addition to these, which regulate the proliferation and migration of keratinocytes, fibroblasts, and VSMCs, because targeting these novel factors may have therapeutic potential. We searched the literature and have listed such factors in the next section.

## 5. Novel Factors Regulating the Proliferation and Migration of Keratinocytes, Fibroblasts, and VSMCs

IL-17 secreted by CD4+ and CD8+ T cells and γδ T cells plays a critical role in the proliferation and migration of epidermal keratinocytes [103], fibroblast proliferation, activation, function (ECM production), senescence, and differentiation [104], and stimulates smooth muscle cell migration involving MMP-9, p38 MAPK and ERK1/2-dependent NF-κB, and AP-1 activation [105]. IL-17 promotes VEGF-dependent angiogenesis in cancer cells [106] and increases the secretion of the angiogenic factors IL-6, IL-8, and VEGF [107]. IL-17 promotes keratinocyte proliferation and enhances wound healing in the early phase of healing while contributing to persistent inflammation and delayed wound healing [108,109]. IL-17 in conjugation with IL-22 delays wound healing in infected skin [110], promotes HIF-1α in wound healing [111], and promotes epithelialization, fibroblast differentiation to myofibroblasts, and keratinocyte activation [112] during wound healing. These studies suggested that IL-17 not only regulates the proliferation and migration of fibroblasts, keratinocytes, and VSMCs but is also involved in regulating inflammation and epithelialization.

Src-kinase-associated protein 2 (Skap2), which is expressed in immune cells, regulates cell migration and motility, immune cell function, and integrin signaling [113]. Integrin signaling is involved in the inhibition of dermal fibroblast migration under hyperglycemic conditions involving KRT17 [114]. In a newly diagnosed patient with type 1 diabetes, Skap2 regulates β-cell apoptosis, controls glucose levels [115], and regulates proliferation, migration, and chemotaxis in macrophage precursor cells. Skap2 is involved in inflammatory diseases and attenuates/prevents excess inflammation via TLR4-NF-κB pathway activation [116]. Skap2 is also involved in promoting angiogenesis by phosphatase and tensin homolog (PTEN) ubiquitination and degradation induction [117]. These findings suggest that Skap2 may play a role in wound healing by regulating angiogenesis, inflammation, and immune cell infiltration.

Phosphodiesterases (PDEs) regulate cyclic nucleotides, which are essential in VSMC proliferation and migration. The reduction of PDE1, by inhibition or deficiency, reduces SMC migration and proliferation. PDE1C has been shown to play a key role in regulating growth factor receptors. PDGF receptor beta is regulated by PDE1C [118]. The inhibition of PDE5 has proven beneficial in wound healing by preventing cGMP degradation and providing high tissue levels [119]. Further, the supplementation of serum with PDE4 inhibition promotes macrophage recruitment for efficient pathogen clearance, promoting wound healing [120]. PDE4 inhibition is also associated with TNF-α and neutrophil elastase-induced fibroblast-mediated collagen gel degradation [121]. These findings suggest that PDEs may be involved in the regulation of multiple mechanisms during wound healing and thus must be investigated.

Factors involved in the proliferation of keratinocytes, fibroblasts, and VSMCs that have yet to be observed regarding diabetic foot ulcers should be utilized in future studies. The N-terminal form of the amyloid precursor protein (sAPPalpha), an epidermal growth factor, promotes the proliferation, migration, and adhesion of keratinocytes [122]. Since amyloid precursor-like protein 2 (APLP2) expression is upregulated in healing the corneal epithelium and promotes healing [123], the possibilities of sAPPalpha promoting healing in wound healing should be investigated. This becomes more important in the context of DFU healing because amyloid presence is associated with poor glycemic control and a higher body mass index [124].

Elastic microfibril interface–located protein 1 (EMELIN1)-α4/α9 integrin is associated with dermal fibroblast and keratinocyte proliferation inhibition in healthy epithelial cells [125]. EMILIN1 is involved in ECM deposition in osteoblasts involving fibulin-4 [126] and ECM remodeling is involved in DFU healing. Further, EMILIN1, a component of elastic fibers that connects it with collagen fibers, which are both important in ECM formation, also regulates cell behavior, growth factor activity, and ECM assembly [127,128], thus the role of EMELIN1 should be investigated in the context of DFU healing. Further, an association of EMILIN1 with islet regeneration [129] supports its probable role in DFU healing by controlling hyperglycemia.

Neuralized E3 ubiquitin protein ligase 1 (NEURL1) is a positive regulator of the Notch pathway. It utilizes E3 ligase activity to promote ubiquitination [130]. Notch is a cell surface receptor involved in proliferation and cell signaling. Hyperglycemic environments play a role in the activation of the notch receptor and contribute to the increased secretion of pro-inflammatory cytokines from macrophages [131]. Since NEURL1 is a positive regulator of the Notch receptor and Notch signaling may be a therapeutic target in DFU healing, further research on the role of NEURL1 in diabetic foot ulcers and as a therapeutic target should be investigated. This notion is supported by the fact that increased Dll4-Notch1 signaling impairs wound healing in DFU [132]. Further, the regulation of adult β-cell proliferation and maturity by Notch signaling makes it worth investigating the role of NEURL1–Notch signaling in promoting DFU healing.

Shisa2 expression, an endoplasmic reticulum (ER) localized protein, is attenuated by Notch signaling [133]. The overexpression of Shisa2 inhibits the proliferation of myoblasts. Shisa2 was found to be upregulated and differentially expressed in type 1 diabetes [134] and may serve as a biomarker. Shisa2 is also a biomarker of pancreatic bud development [135], the organ related to diabetes. Shisa2 is a negative regulator of Wnt signaling and is related to the inflammatory response [136], as well as to ferroptosis in airway epithelium in patients with asthma [137]. Since inflammation plays a critical role in DFU pathogenesis and Wnt signaling is involved in DFU pathogenesis, the role of Shisa2 should be investigated in the context of DFU healing.

Cerebral cavernous malformation (CCM)2, which functions in the stress-activated p38 Mitogen-activated protein kinase (MAPK) signaling cascade, is an adaptor protein called malcavernin that strengthens endothelial cell junctions and stabilizes vessels. The loss of CCM2 knockdown reduces EC migration and attenuates wound healing [138] and endocardial growth factor in wound healing [139]. A paralog of CCM2 (CCM2L) delays wound healing in mice by attenuating angiogenesis [140], while in cardiac cells, CCM2L promotes cardiovascular cell growth [139]. Endothelial cells (ECs) with silenced CCM1 and CCM2 change their phenotype to enter a senescence-associated secretory phenotype (SASP), which ECs use to invade the ECM and attract surrounding wild-type ECs and immune cells [141]. Angiogenesis is important in wound healing and CCM proteins regulate VEGF-mediated angiogenesis via the β1 integrin-Klf2-Egfl7-signaling pathway [142]. It should be noted that CCM2 and CCM3 differentially regulate the response of ECs and angiogenesis [143] in central cavernous malformation. CCM2 regulates RhoA activity to maintain vascular integrity and the knockdown of CCM2 is associated with impaired EC migration [138]. These studies suggest that CCM proteins may play a critical role in angiogenesis during wound healing by regulating EC migration, which is involved along with VSMCs in angiogenesis. Investigating the role of CCM in DFU healing is important because long-term treatment with high glucose attenuated CCM1 expression [144].

Ladybird homeobox 1 (*Lbx1*), a protein-coding gene, is necessary in lateral muscle migration and regulates the response to lateral migration signals in limb development [145]. *Lbx1* is involved in neuronal cell fate determination [146] and myoblast cell proliferation for myogenesis [147], suggesting its probable role in wound healing because myogenesis and arteriogenesis enhance wound healing [148] and myofibroblasts can be reprogrammed to adipocytes during wound repair [149]. Thus, an increased population of myofibroblasts at wound sites, whose proliferation and migration are regulated by *Lbx1*, may have a role in promoting wound healing though to be invested.

Shroom3, a PDZ-domain protein, identified in kidney tissues, is required in epithelial redifferentiation and repair. Shroom3 has also been shown to play a role in proliferation and myofibroblast activity [150]. Shroom3 regulates the epithelial cell shape, involving the apical positioning of the actomyosin network [151]. *Shroom3* involves cytoskeletal architecture regulation and maintenance, is expressed in VSMCs, and spatially has a similar expression to α-smooth muscle actin (α-SMA). The downregulation of *Shroom3* expression is associated with VSMC hypertrophy [152]. Not directly related to the proliferation and migration of fibroblasts, keratinocytes, and VSMCs; the regulation of myofibroblast activity, epithelial cell shape, and VSMC hypertrophy suggest that *Shroom3* may play a role in wound healing but warrants in-depth investigation.

Strip2, a member of the striatin-interacting phosphatase and kinase complex, promotes the proliferation and migration of VSMCs involving the P38 MAPK-AKT-MMP-2 signaling pathway [153]. The silencing of Strip2 is associated with abnormalities in vascular and heart development [154]. Strip2 has been identified as a key regulator in the differentiation of embryonic stem cells (ESCs) [140,154]. Further, stem cells are beneficial to wound healing in DFUs, by enhancing wound healing and promoting angiogenesis [28]. Further, stem cells, whose differentiation is regulated by Strip2, play a critical role in the migration and proliferation of fibroblasts towards the wound site, promoting collagen production and skin wound healing [155].

The novel factors discussed above suggest that these factors play a role in the proliferation and migration of keratinocytes, fibroblasts, and VSMCs, and angiogenesis, vascular stability, and wound healing; however, their direct role in DFU healing is uncertain. Thus, there is a need to investigate their role in DFU healing and this should be the focus of future research to determine their potential role. Further, it should be confirmed how these factors alone or in combination may contribute to DFU healing from the perspective of previous studies. To find the interaction between these genes and their association with the various mechanisms involved in DFU healing, we performed a network analysis using the STRING network and Networkanalyst.ca, with the input genes discussed above.

## 6. Network Analysis

The network analysis using the STRING network revealed an interaction between various proteins, including TGFB1, THBS1, MMP2, EGF, IGF1, MMP9, TNF, PDE4A, PDE5A, IL17A, CXCL8, IL8, IL10, IL1B, CD40, PTEN, TLR4, IL6, and others, suggesting their interactive role. For instance, TGF-β1 is involved in keratinocyte proliferation, migration, differentiation, and phenotypic switch [59,60], TGF-β3 induces regenerative characteristics in dermal fibroblasts [62], and TGF-β1 promotes the proliferation and migration of fibroblasts during wound healing [63]. Thrombospondin-1 (THBS1), a matrix glycoprotein, activates TGF-β1 and affects fibrosis by promoting the proliferation and migration of hypertrophic scar fibroblasts [156]. MMP-2 and -9 stimulate keratinocyte, fibroblast, and endothelial cell proliferation and migration and promote new granulation tissue and wound healing [157]. Phosphodiesterase regulates the proliferation, migration, and differentiation of various cells, including epithelial cells, and plays a role in EMT [158], which in turn plays a critical role in DFU healing. The role of CD40 in DFU healing and fibroblast phenotype changes has been reported in our previous study [13]. Further, various cytokines, including IL-1β, TNF, IL-17, IL-22, and IL-8, and pattern recognition receptors (TLRs) play a critical role in keratinocyte proliferation, inflammation regulation, and immune response [159], all playing a critical role in wound healing. The role of cytokines during wound healing is further supported by their changing levels after electromagnetic field stimulation [160], which promote wound healing after injury [161]. An interaction between these factors suggests their regulatory role in wound healing.

**Figure 2:**
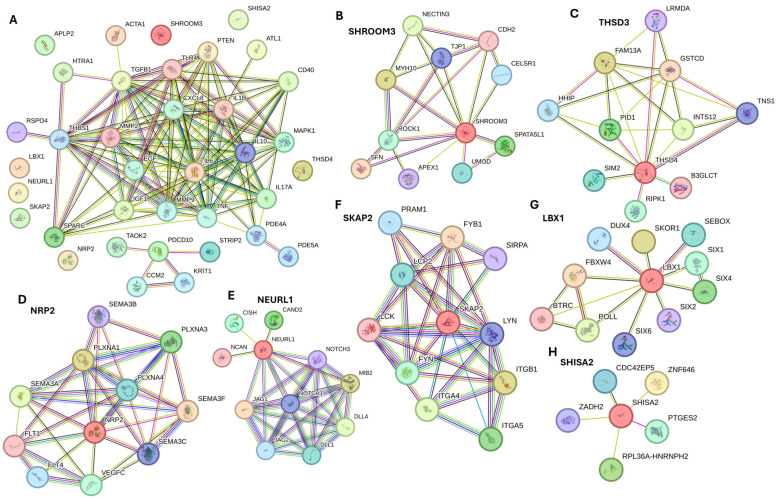
Network analysis of various proteins using the Search Tool for the Retrieval of Interacting Genes/Proteins (STRING) network to predict functional associations between different proteins. (**A**) STRING network interaction of input gene list showing protein-protein interaction, (**B**) interactions for SHROOM3, (**C**) interactions for THSD3, (**D**) interactions for NRP2, (**E**) interactions for NEURL1, (**F**) interactions for SKAP2, (**G**) interactions for LBX1, and (**H**) interactions for SHISA2.

However, the STRING networking did not reveal the interaction of APLP2, SHROOM3, SHISA2, NEURL1, LBX1, and NRP2 with others (Figure 2A). Thus, we searched each protein in the STRING network and found associations of these proteins with factors contributing to various mechanisms involved in wound healing.

SHROOM3 (Figure 2B) showed an interaction with CDH2, which along with CDH11, plays a significant role in wound healing during the phenotypic change of fibroblasts to myofibroblasts, contributing to wound contraction and closure [162]. Thrombospondin type-1 domain-containing protein 4 (THSD4) (Figure 2C) was found to be upregulated with regeneration involving TGF-β signaling [163]. Neuropilin 2 (NRP2), a coreceptor that enhances human endothelial cell biological responses induced by VEGF-A and VEGF-C, showed an association of SEMA3F, SEMA3C, SEMA3A, SEMA3B, and VEGFC (Figure 2D), suggesting its involvement in regulating angiogenesis during DFU healing [11] and wound healing under hyperglycemic conditions [164]. Neuralized E3 Ubiquitin Protein Ligase 1 (NEURL1) (Figure 2E) showed an interaction with Notch1, Notch3, DLL1, and DLL4. NEURL1 accelerates wound healing in the corneal epithelium [165]. Notch signaling plays a critical role in cell differentiation, proliferation, and angiogenesis during DFU healing [132]. Src-kinase-associated protein 2 (SKAP2), an adaptor protein involved in integrin signaling, showed an interaction with ITGB1, ITGA5, and ITGA4 (Figure 2F), which are involved in diabetic ulcer healing [166,167]. LBX1 revealed interactions with SIX1, SIX2, SIX4, SIX6, and DUX4 (Figure 2G). The SIX family of genes/transcription factors are involved in the proliferation of muscle satellite cells [168] and regulate angiogenesis [169]. SHISA2 (Figure 2H) showed an interaction with zinc-binding alcohol dehydrogenase domain-containing 2 (ZADH2/PTGR3), which negatively regulates adipose/fat cell differentiation [170]. ZADH2 has been reported to have similar expression levels to VEGFA in gene expression studies [171], and since VEGFA is essential for angiogenesis, ZADH2 may also play a role.

**Figure 3:**
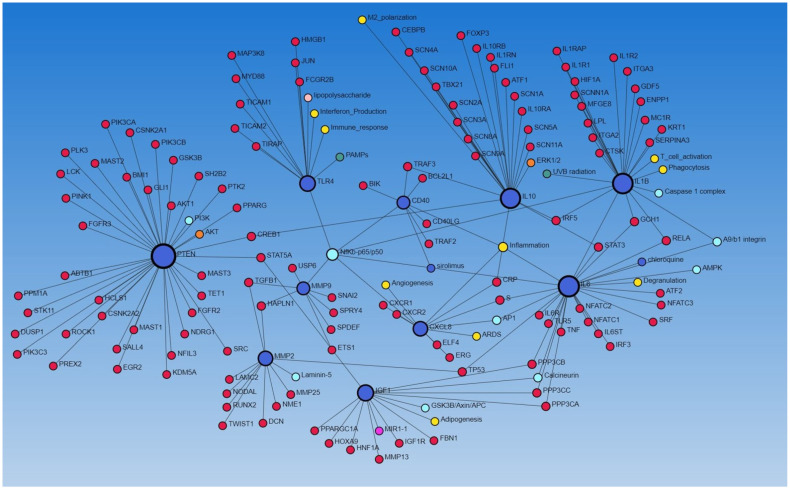
Network analysis between different proteins using the STRING database via NetworkAnalyst.ca.

To further establish the interaction between various genes (encoding proteins), we performed a network analysis using NetworkAnalyst.ca (STRING and IMEx database). The network analysis using the STRING database revealed the interaction of IGF1, MMP2, MMP9, IL6, PTEN, TLR4, CD40, IL10, CXCL8, TP53, TGFB1, and IL1B (all involved in regulating wound healing as discussed above) with each other, as well as with other proteins, and the molecular/biological process, including angiogenesis, adipogenesis, inflammation, M2 differentiation, T-cell activation, phagocytosis, immune response, integrins, and interferon production (Figure 3), suggesting their role in diabetic ulcer healing. We have previously reported the increased expression of the CD40+ fibroblast population in DFU contributing to inflammation [13].

**Figure 4:**
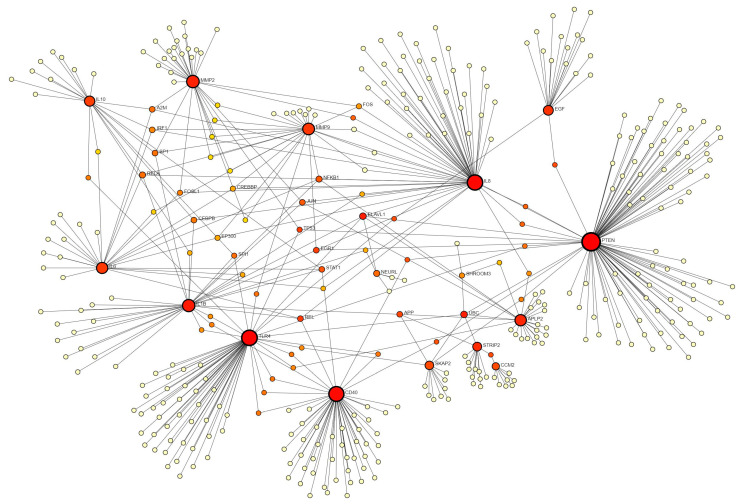
Network analysis using the IMEx database in Netwrokanalyst.ca.

Using the STRING database did not reveal the interaction between various proteins (encoded by the genes discussed above) and hence we used the IMEx database. The analysis revealed an interaction between PTEN, EGF, IL8, MMP2, MMP9, APLP2, STRIP2, CCM2, IL6, IL1B, SPI1, STATs, IRF1, SP1, RELA, JUN, FOS, NFKB1, CEBPB, EP300, IL10, CD40, and TLR4 (Figure 4), playing a critical role in wound healing. The regulatory role of EGF, IL-8, MMP-2, MMP-9, IL1B, IL-10, CD40, and the TLRs has been discussed above. PTEN (phosphatase and tensin homologue) dephosphorylates PIP3 (phosphatidylinositol(3,4,5)-trisphosphate) and negatively regulates PI3Ks (phosphoinositide-3 kinases). PTEN inhibition promotes epithelial wound healing by significantly increasing the level of phosphorylated Akt (protein kinase B) [172] and keratinocyte proliferation [173]. Keratinocyte proliferation, adhesion, and migration are impaired by APP/APLP2 deficiency [122]. *CCM2* gene codes for the protein malcavernin, which strengthens the interactions between the cells involved in blood vessel formation, thus its decreased expression may lead to leaky vessels or impaired angiogenesis [174]. STRIP2 interactions with other factors, including CCM2, UBC, SHROOM3, and APC, suggest its probable role during wound healing; however, the role of STRIP2 in wound healing in the existing literature is not defined.

The interaction between and with other proteins (Figure 4) and with some of the proteins that appeared in other networks (Figure 2 and Figure 3) suggests that the novel factors discussed above may play a role in the proliferation and migration of epithelial cells, fibroblasts, and VSMCs, along with other molecular mechanisms involved in the healing of diabetic foot ulcers. However, these interactions and their role alone or in combination in promoting DFU healing warrant investigations.

## 7. Translational Significance of Novel Factors and Factors in the Network Analysis

The description of the novel factors listed in Section 5 and Section 7 and their interactions with each other and the other factors involved in wound healing suggest their probable role in the pathogenesis of DFU and their suitability as therapeutic targets. However, it is important to relate these factors to diabetes to emphasize their role in DFU. For instance, IL-17 induces the expression of pro-inflammatory cytokines and chemokines, deteriorates β cell function, and induces insulin resistance in diabetes [175]. Skap2 regulates β-cell apoptosis and controls glucose levels [115]. PDE4 dysregulation plays a critical role in metabolic syndrome and PDE4 inhibitors attenuate diabetes symptoms and improve insulin resistance and hyperglycemia [176]. PDE5 inhibitors may act as insulin sensitizers [177]. Amyloid presence is associated with poor glycemic control and a higher body mass index [124]. APLP2 regulates glucose and insulin levels. Neuritin promotes neurite outgrowth and synapse maturation during neural development and regeneration and inhibits NEURL1, a regulator of Notch signaling [178]. Since diabetic neuropathy plays a critical role in DFU pathogenesis, the roles of NEURL1 and neuritin are worth investigating. Shisa2 was found to be upregulated and differentially expressed in type 1 diabetes [134] and may serve as a biomarker. Diabetes-induced hypermethylation inhibits *Shroom3* and suppress neural tube closure [179]. *Shroom3* also contributes to the maintenance of the glomerular filtration barrier integrity [180], which is altered in diabetes. An interaction of these factors (involved in diabetes pathogenesis) with PTEN, EGF, IL8, MMP2, MMP9, APLP2, STRIP2, CCM2, IL6, IL1B, SPI1, STATs, IRF1, SP1, RELA, JUN, FOS, NFKB1, CEBPB, EP300, IL10, CD40, and TLR4 plays a critical role in DFU pathogenesis, as discussed above, and the network analysis suggests that these factors may be therapeutic targets in DFU treatment. This notion is supported by the fact that targeting inflammation [181], ECM remodeling [182], and MMP-9 [183] to promote DFU healing have been suggested in the literature. Further, the network analysis showed an interaction between IL-8 and APLA2 and an increased expression of CXCL8 in nonhealing DFU, and IL-8 as a suitable target to promote healing in DFU has been proposed [8,184].

## 8. Conclusions

The proliferation, migration, and differentiation of fibroblasts, keratinocytes, and smooth muscle cells play a critical role in normal wound healing, as well as in the healing of diabetic foot ulcers (DFUs). However, in the diabetic wound environment, the normal progression of healing is disrupted due to persistent inflammation. The factors governing the proliferation and migration of these cells significantly influence the stages of wound healing and are crucial in understanding the mechanisms of DFU nonhealing. The novel factors discussed in this review highlight promising avenues identified through a network analysis, suggesting their potential role in promoting wound healing alone or in combination. However, to establish their role in wound healing, further research and clinical trials are warranted, mainly in the context of a hyperglycemic environment to establish better therapeutics for chronic nonhealing DFUs.

## Figures and Tables

**Table 1:** Role of growth factors in regulating various mechanisms during wound healing.

Growth Factor	Normal Wound Healing	Diabetic Wound Healing
IGF-1	Stimulates proliferation of many cell types, including fibroblasts in association with other growth factors, such as PDGF or FGF	Keartinocyte proliferation and migration, re-epithelialization, and angiogenesis
FGF (bFGF, FGF-7, FGF-10, FGF-2)	FGF-2 secretion increases during acute wound healing and enhances granulation tissue formation, re-epithelialization, and tissue remodelling. FGF-2 increase keratinocyte motility and the production of ECM components.	Targets keratinocytes, fibroblasts, endothelial cells (bFGF), and keratinocytes (FGF-7 and FGF-10). Regulates angiogenesis, granulation tissue formation (bFGF), re-epithelialization (FGF-7), and the detoxification of ROS (FGF-10).
IGF-1 * EGF	Stimulates the proliferation and migration of all types of epithelial cells	Tissue formation and re-epithelialization by targeting fibroblasts, endothelial cells, and keratinocytes
VEGF	Promotes angiogenesis by acting on endothelial cells	Regulates angiogenesis and inflammation by targeting endothelial cells and macrophages
TGF-β	TGF-β1 stimulates collagen deposition, inhibits collagen degradation, and regulates inflammation. TGF-β3 inhibits scar formation.	Targets fibroblasts, keratinocytes, macrophages, leukocytes, and endothelial cells to regulate granulation tissue formation, collagen synthesis, matrix formation and remodeling, inflammation, leukocyte chemotactic function, and angiogenesis
* PDGF	Initiates the inflammatory response by the chemotaxis of neutrophils, macrophages, fibroblasts, and smooth muscle cells. Increases the secretion of IGF-1 and Thsp-1 contributing to re-epithelialization.	Regulates inflammation, re-epithelialization, collagen deposition, and tissue remodelling by targeting leukocytes, macrophages, and fibroblasts
HGF	Promotes the dedifferentiation of epidermal cells	Targets endothelial cells, keratinocytes, and regulates inflammation (attenuation), granulation tissue formation, angiogenesis, and re-epithelialization

Hepatocyte growth factor (HGF), insulin-like growth factor (IGF), fibroblast growth factor (FGF), epidermal growth factor (EGF), vascular endothelial growth factor (VEGF), transforming growth factor beta (TGF-β), and platelet-derived growth factor (PDGF). * indicates the growth factors with clinical trials in human patients (details are in Table 2).
